# Probabilistic Approach to Robust Wearable Gaze Tracking

**DOI:** 10.16910/jemr.10.4.2

**Published:** 2017-11-08

**Authors:** Miika Toivanen, Kristian Lukander, Kai Puolamäki

**Affiliations:** a Finnish Institute of Occupational Health, Helsinki, Finland; b University of Helsinki, Helsinki, Finland

**Keywords:** Wearable gaze tracking, Human eye modeling, Bayesian modeling, Kalman filtering

## Abstract

This paper presents a method for computing the gaze point using camera data captured with a wearable gaze tracking device. The method utilizes a physical model of the human eye, advanced Bayesian computer vision algorithms, and Kalman filtering, resulting in high accuracy and low noise. Our C++ implementation can process camera streams with 30 frames per second in realtime. The performance of the system is validated in an exhaustive experimental setup with 19 participants, using a self-made device. Due to the used eye model and binocular cameras, the system is accurate for all distances and invariant to device movement. We also test our system against a best-in-class commercial device which is outperformed for spatial accuracy and precision. The software and hardware instructions as well as the experimental data are published as open source.

## 1 Introduction

Gaze tracking is finally evolving from studies that are mainly performed in controlled laboratory environments and desktop trackers to field studies running on wearable hardware. These kind of mobile systems are applicable to actual operational environments and workplaces, e.g., walking, driving, operating machinery, and interacting with the environment and other people. Mostly due to the novelty and high price of the mobile gaze tracking technology, application potential remains, for the most part, unutilized.

Current commercial systems for wearable gaze trackers offer working solutions for industrial and research customers but are expensive and hide the implementation of their tracking hardware and algorithms behind proprietary solutions. This limits their accessibility, areas of application, and the scale and scalability of gaze tracking and inhibits their further development, integration, customization, and adaptation by the expert community of gaze tracking users and researchers. Also, the reported performance metrics are hard to cross-evaluate between systems as they are typically reported for “optimal” tracking conditions and well-performing subjects. Therefore, while commercial systems can supply high-performing solutions, there is still a need for a low-cost alternative that opens up the further development of gaze tracking methods and algorithms. Enabling the larger user community to build their own trackers opens the playground for innovation, novel approaches, and more rapid development cycles. Computationally, the big challenges in mobile gaze tracking include changing lighting conditions and movement of the device in relation to the eyes of the users.

In this paper, we present the algorithmic basis of our current open-source gaze tracker. The system provides a robust, probabilistic approach for realtime gaze tracking, especially for use with a mobile frame-like system, although the presented algorithms should also work for remote settings. The algorithms are based on advanced Bayesian modeling while utilizing a physical eye model. The system has proven very robust against dynamic changes in external lighting and possible movement of the gaze tracking frame in relation to the eyes of the user.

Our hardware implementation is built from off-the- shelf sub-miniature USB cameras and optical filters, a simple custom printed circuit board, and a 3D printed frame. Apart from a standard laptop computer for running the software, the total cost for the system is ca. 700 euros of which 80 % is dictated by the cameras. The software and the design of the circuit board and frame are released as open source under a permissive license.

The temporal requirement of realtime systems (i.e., processing the video frames within the frame rate of the cameras) poses a challenge. Conventional USB-cameras capture 20-30 frames per second which our software implementation is able to handle. However, due to the complexity of the methods, using cameras with higher frame rates (such as 100 fps) may call for special solutions such as hardware acceleration or parameter optimization.

In addition to performance metrics, we provide a comparison to a best-in-class commercial system (SMI Eye- tracking Glasses with iView 2.1 recording software and BeGaze 3.5 analyzing software) in the same setting. Based on our experiments with 19 participants, the commercial system is outperformed for spatial accuracy and precision. The recorded data is published openly, too.

Summarizing, the contributions of this paper are:

• A complete probabilistic gaze tracking model and its full implementation.
• Exhaustive evaluation of the performance of our system and publication of the recorded data openly for others to test.
• Validation of the performance of the commercial SMI gaze tracking glasses system.
• C++ software implementation and hardware instructions published as open source.

### 1.1 Related Work

The large majority of gaze tracking research thus far has been performed in desktop environments using remote trackers, with the eye tracker and light sources integrated to the desktop environment and calibrated for a planar computer monitor. A number of wearable gaze trackers, both commercial and open-source, are also available. These allow the user to move around more freely and track gaze outside a single screen space. While the basic tracking methodology utilized is largely similar - tracking optical features of the eye - there is large variation in technical details, performance, and robustness between the systems. A wide survey of gaze tracking methodology is presented by Hansen and Ji (8). Hayhoe and Ballard (9) offer a more mobile-centric review and Evans et al. (4) focus on outdoor gaze tracking and some of the complexities inherent in taking gaze tracking out of the lab.

The most typical solution in video-based gaze tracking is to track the pupil and usually one corneal reflection (CR) from a light source to offer rudimentary compensation for camera movement relative to the eye. These methods use a multi-point calibration on a fixed distance, mapping changes in pupil and CR positions to interpolated gaze points (Duchowski, 3). A more sophisticated solution utilizing a physical model of the eye was suggested in the desktop genre (Shih and Liu 22; Guestrin and Eizenman 6; Hennessey et al., 10). These require at least two light sources and the respective CRs for solving the geometrical equations involved and also some sort of user calibration to compensate for the personal eye parameters. User calibration is usually lighter than with mapping methods; Chen and Ji (1) even proposed a calibration-free model based method.

The mobile setting poses more challenges than the desktop setting due to, e.g., changes in lighting and device orientation. As an added complication, the gaze distance varies when the user freely navigates her environment which leads to variable parallax error when gazing at different distances as the eyes and the camera, imaging user’s view, are not co-axial (Mardanbegi and Hansen, 18). The gaze distance can be approximated with at least three solutions: use a fixed distance, use metric information about the environment based on visual (fiducial) markers, or employ binocular trackers (i.e, having eye cameras for both eyes) and produce an estimate for gaze distance using the convergence of both eyes’ gaze vectors that can be computed with the physical model.

Commercial systems offer proprietary solutions to gaze tracking. Notable examples include Tobii Pro Glasses *(http://www.tobiipro.com/product-listing/tobii-pro- glasses-2/)*, Ergoneers Dikablis system *(http://www.er- goneers.com/en/hardware/eve-tracking/eye-tracking- head-mounted/)*, and SMI Gaze tracking glasses *(http://www.eyetracking-glasses.com/)*, the latter used here as a reference for evaluating performance. A considerable number of open solutions for gaze tracking have also been suggested. Earlier mobile open source gaze tracker systems include the openEyes system (Li, Bab- cock, and Parkhurst, 16) that introduced the popular Starburst algorithm for detecting eye features; the ITU Gaze Tracker system (San Agustin et al., 20) that aims at providing a low-cost alternative to commercial gaze trackers; and the Haytham (Hales, Rozado, and Mardanbegi, 7), developed more toward direct gaze interaction in real environments. Pupil labs offer both commercial and open-source systems (Kassner, Patera, and Bulling, 14) and Ryan, Duchowski, and Birchfield (19) aimed at a tracker operating under visible light conditions.

Here, we extend the physical eye model approach introduced by Hennessey, Noureddin, and Lawrence (10). We utilize Bayesian methodology to provide a robust method for accurate gaze tracking in real time. Work toward the current system has been described by Lukander et al. (17) which used a similar approach but with a different optical setup, monocular tracking, and a heuristic feature tracking solution; Toivanen and Lukander (26) who presented probabilistic ideas for tracking the eye features; and Toivanen (24) who introduced a preliminary version of the Kalman filter for stabilizing the result.

## 2 Proposed method

Before going into more details with the method, let us give some definitions. The method is supposed to be used with a wearable gaze tracking system, a.k.a., *gaze tracking glasses*. The glasses contain one or two *eye cameras* that point towards eyes. There is also a *scene camera* pointing towards user’s scene. The glasses contain LED light sources, attached to the frame of the glasses. Each LED causes a reflection on the eye surface as seen by the eye camera, called a *glint*. The mutual configuration of the glints is specific to the placement of the LEDs in the glasses although this configuration changes according to the shape of the eye and pose and distance of the glasses with respect to the eye. The (average) mutual configuration, or shape, of the glints is called a *glint grid*. The number of LEDs is denoted with *N_h_* and the glint grid contains thus *N*_l_ glints. The captured eye image is generally denoted with *J* throughout the paper but its specific form (in terms of preprocessing) depends on the context and is clarified accordingly. An example of gaze tracking glasses with six infrared (IR) LEDs is shown in Figure 11, and an example of an eye image, captured with it, is given in Figure 2. For the sake of clarity, throughout the paper the notation for matrices and vectors is not bolded except when being multivariate.

The objective of the presented method is to estimate the three-dimensional point-of-gaze (POG) and its 2D projection in the image plane of the scene camera, utilizing a simplified eye model and knowledge about the configuration of the cameras and LEDs in relation to each other. The identified 3D POG can also be projected to other reference coordinate systems, such as one based on fiducial markers detected in the scene image, but here we concentrate on the case of scene video.

Mobile gaze tracking sets additional requirements for the performance as compared to desktop gaze trackers: dynamic lighting conditions require better tolerance for changing luminosity and extra reflections and tracking the eyes of a moving subject calls for robustness against possible movement of the device in relation to the tracked eyes. This necessitates using more LEDs than what is typically used in desktop trackers which then again increases the probability of some glints being non-visible. Also, in wearable systems the LEDs must be located at the very edge of the view to minimize their disturbance whereas in remote systems the LEDs can be located near center of the field of view. In addition, with the desktop gaze trackers the gaze distance is approximately constant, as opposed to mobile tracking. While a simpler mapping-based approach may be sufficient for desktop trackers, the variable nature of mobile tracking is better handled with a model-based approach.

### 2.1 System overview

**Figure 1:**
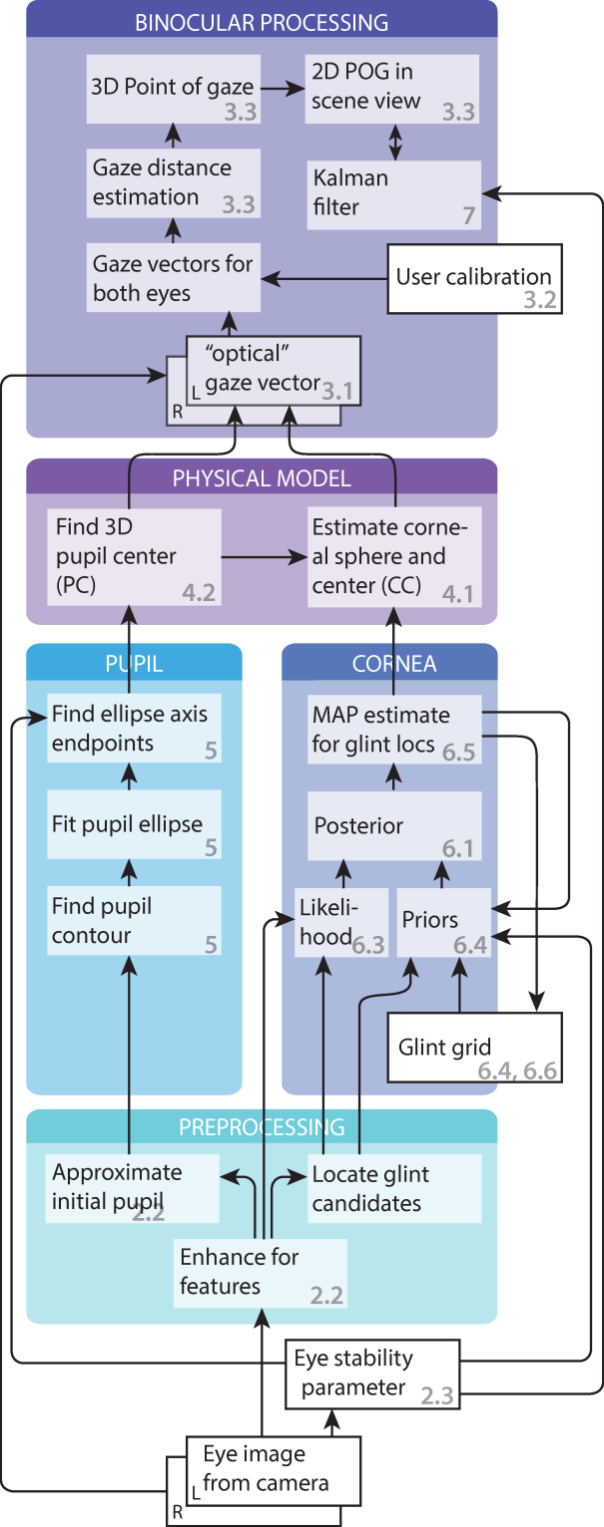
A flowchart of the gaze tracking solution. The numbers refer to sections and subsections where the item is described.

Figure 1 presents a flowchart of the processing pipeline for video frames. The frames grabbed from the cameras are first preprocessed for finding the pupils and the corneal features. These are used in computing the 3D cornea centers and pupil locations of the physical eye model. From these, the gaze vectors, and ultimately the gaze point in the scene video can be computed utilizing user calibration information. To stabilize the results, the gaze point can further be Kalman filtered. Here, the focus is on supplying gaze location and path. However, fixations and saccades can be roughly estimated using the eye stability parameter which estimates how stable the eye has been during the latest video frames. In addition, the frames where eye features cannot be detected can be assigned as blinks.

For the utilized physical eye model (“physical model” block in the flowchart) we use the model presented by Hennessey (10); Shih and Liu (22). The main contribution of this paper arises from applying the method to wearable gaze tracking and the methodology in the other blocks in Figure 1. Probably the greatest contributions are in the computer vision related tasks (“pupil” and “cornea” blocks) and in the Kalman filter. The glint grid is generally more difficult to locate than the pupil because there may be additional distracting reflections in the image and some of the glints may not always be visible. Therefore, we use a simpler detection scheme for pupil and a more advanced Bayesian tracking model for glints. The Bayesian approach allows neatly combining the expectations about the appearance of the glints and their mutual configuration. In addition, glints are likely to depend on their locations in the previous image; for most of the time, eyes fixate on the same point and the glints are stationary. With the Bayesian tracking framework, we can utilize this information in a sophisticated manner.

The cameras are modeled as pinhole cameras. The intrinsic and extrinsic parameters are estimated using the conventional calibration routines (Zhang, 27).

### 2.2 Preprocessing the eye image and approximating the pupil center

**Figure 2:**
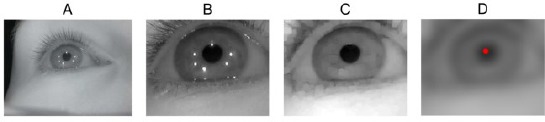
An example of a captured eye image (A) which is cropped (B), morphologically opened (C), and filtered (D). The filtered image shows also the estimated pupil center which is used later in estimating the glint locations and pupil edges.

Figure 2A illustrates an eye camera image captured while wearing the glasses that are shown in Figure 11. The pupil and all the LED reflections (i.e., glints) are clearly visible. The eye images are preprocessed to decrease the image size by cropping away useless parts and color channels in the image in order to accelerate the computations, and to remove noise and enhance certain image features. Images B, C, and D of Figure 2 shows some preprocessing stages.

The captured IR image is practically monochromatic so it is first converted to a grayscale image. Because the eye can be assumed to stay at a relatively fixed position in the image, the image is cropped around the assumed eye location, using a constant cropping area (B). For finding the initial approximation for the pupil, the cropped image is morphologically opened to remove the glints (C). Then, an approximate pupil center is located in order to further crop the image. For estimating the pupil center, the opened image is filtered by convolving it with a circular kernel whose radius approximately equals the expected radius of a pupil in the eye images; this removes dark spots that are smaller than the pupil so that after the filtering the pupil center would be the darkest pixel, which is considered to be the approximate pupil center (D). Despite the relative simplicity, this approximate pupil center detection performs well. In our experiments, only very dark and thick make-up in the eyelashes caused the pupil center to be misdetected in the eyelash area.

Different preprocessed stages are used in different parts of the algorithms: the eye stability estimator uses the filtered image (Figure 2D); the pupil localization uses the opened image (C) and estimated pupil center; and the glint localization uses the cropped and opened images (B and C) together with the estimated pupil center.

### 2.3 Estimating stability in eye image

We use information about how stable the eye image has been during the most recent *k + 1* previous video frames later in Sections 5, 6.4, and 7. To quantify this stability measure we use a simple scaled and smoothed estimator of an average change in the eye images that gives a value *θ ∈* [0,1] which is close to unity if there have been large changes in recent frames and close to zero if the eye seems to have been relatively stable.

More formally, given parameters 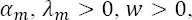, and the size of the smoothing window *k +* 1, we define the stability *9^t^* at time instance *t* as


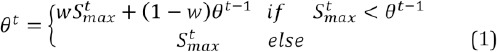


where 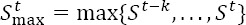 is the maximum value of the previous *k + 1* sigmoid values, defined as


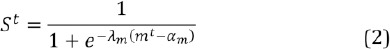


where *m^t^* is a pixel-wise *L_2_* norm between subsequent frames:


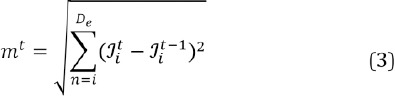


where J^t^ is the observed (cropped, opened, and filtered) eye image at time *t* (see Figure 2D for an example) and *D_e_* is the number of pixels in the image. The parameter *θ^t^* is estimated for the right eye image only since the eyes normally move in synchrony. The parameter values that we use in our experiments are presented in Table 2.

**Table 1:** The anomalies of the dataset

ID#	Issue
06	Has heavy mascara
07	Some of the calibration dots were not visible
11	Calibration videos of the presented system were destroyed
13	Calibration videos of the presented system were destroyed
13	SMI calibration possibly failed

**Table 2:** The parameters of the model, their explanation, the corresponding equation or section in the text, and the values used.

param.	explanation	in text	value
β	likelihood steepness	Eq.(21)	100
κ	prior covariance regulator	Eq.(27)	1
с	max. variance in dyn. prior	Eq.(29)	100
Q	process variance in Kalman filter	Eq. (39)	1
R_v_	velocity meas. variance in Kalman filter	Eq. (47)	1
R_max_	max. meas. variance in Kalman filter	Eq. (48)	100
λ_m_	sigmoid parameter	Eq. (2)	0.02
α_m_	sigmoid parameter	Eq. (2)	500
W	increase of θ after saccades	Eq. (1)	0.7
T_pr_	number of prior measurements	Eq. (36)	10
γ_min_	minimum θ value in pupil detection	Eq. (16)	0.2
N_gl.cand._	number of glint candidates	Sec. 6.5	6
thold	threshold in pupil detection	Sec. 5	0.2

## 3 Physical eye model

The following section details the basic principles of computing the POG with the physical model.

### 3.1 Gaze point computation

**Figure 3:**
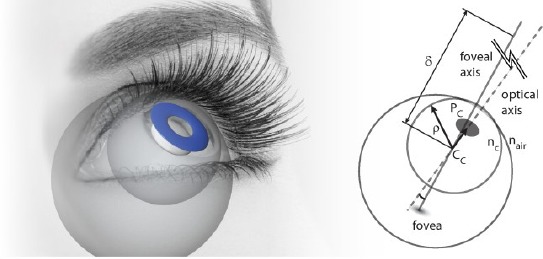
The simplified model of the human eye. The pupil is actually a hole in the iris, allowing light to enter the retina. The corneal surface can be modeled as a sphere partly embedded in the eye. Pc and Cc refer to centers of the pupil and corneal sphere through which the optical axis traverses. The radius of the corneal sphere is denoted with p. nc and nair refer to refractive indices of cornea and air. Fovea is the spot on the retina with the highest density of photoreceptors. The axis that (approximately) traverses the gaze point, Cc, and the fovea is called the foveal axis (to be precise, the visual axis and the optic axis intersect at the nodal point of the eye which is within 1 mm of the cornea center (Guestrin and Eizenman, 6)). δ is the gaze distance along the foveal axis between Cc and gaze point.

Figure 3 illustrates the used (simplified) physical model of the human eye. The axis that traverses the 3D centers of the pupil *(P_c_*) and the corneal sphere *(C_c_*) is called the optical axis. The corresponding unit vector is called the optical vector, denoted as *L*. The actual gaze vector that we are interested in traverses (approximately) through the cornea center and fovea which is the spot on the retina with the highest density of photoreceptors. The optical and gaze vectors are not parallel. Hence, the gaze point *g_p_* is computed as


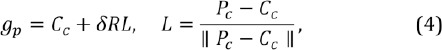


where *R* is a matrix that rotates the optical vector to coincide with the gaze vector and *δ* is the viewing distance between *C_c_* and the gaze point. Note that (4) applies for both eyes, each with its own gaze point. If two eye cameras are in use, the gaze distance *δ* can be estimated (see Subsection 3.3).

### 3.2 User calibration

The rotation matrix *R* is a person-dependent constant as the location of fovea, and thus the angular difference between the optical and foveal axes vary between individuals. This section describes how to estimate *R*; this procedure is called user calibration.

Let us assume that we know the transformation between the coordinate systems of each camera (both eye cameras and the scene camera). The 3D points *P_c_* and *C_c_* are originally defined in the coordinate system of the corresponding eye camera. For computing the gaze point, the 3D points in the left eye camera coordinates are transformed to the right eye camera coordinates. Then we can transform both eye’s gaze point to scene camera coordinates. Let *A* and *a* denote the rotation and translation parts of this transformation. The gaze point in the scene camera, *q*, can thus be computed as


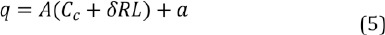


where *C_c_*, *δ, R*, and *L* “belong” to either the left or the right eye.

In the user calibration procedure, the user fixates several points which are a fixed distance *δ* away from the user and which are simultaneously annotated in the scene camera video. For each annotation, the cornea centers and gaze vectors are extracted as well as the annotated 3D target values which can be estimated using the camera calibration information and the fixed distance to the calibration target. The calibration routine with *N_c_* samples results thus in a collection of points and vectors *{*q^i^*, *C^i^_c_*, L^i^, i = 1,2,…, N_c_}* which we denote with *q^cal^*, C_c_^cal^, and *L^cal^*. Eq. (5) should hold for each sample as well as possible so *R* is estimated in a least-squares sense as


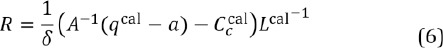


where the inverse of *L^cal^* can be solved with, e.g., pseudoinverse, as long as *N_c_ ≥ 3*. Note that the viewing distance *δ* must be set to the real distance between the viewer and target, which distance is also used for computing the target points *q^cal^* - this should lead to negligible error as it can safely be assumed that the distance from the scene camera origin to the target approximately equals the distance from the cornea center to the target because the target distance is much larger than the distance between the scene camera and the eye. Calibration sets and *L^cal^* naturally differ between the eyes, resulting in separate calibration matrices for both eyes, *R^L^* and *R^R^*.

The matrix *R* is, however, not strictly a rotation matrix but a general transformation matrix. This transformation can be thought of as dealing with all other kinds of (more or less systematic) inaccuracies present, apart from the angle between gaze vector and optical vector. Errors may stem from the following factors: some user-dependent eye parameters are set to constant population averages, the physical eye model is over-simplified, the used pinhole camera model is only approximate (while calibrated), the estimated transformation matrices between LEDs and cameras are imperfect, and there may be inaccuracies in the estimated corneal and pupil centers. Because *R* is not a rotation matrix, its determinant is not unity and it is not orthogonal. Therefore, the unit of *δ* is, strictly speaking, not metric but the norm of *RL* which might slightly deviate from unity and may thus attribute a minor error into the estimation (6). Finally, it should be noted that the user calibration needs to be done only once for each user after which the same calibration information can be used repeatedly.

### 3.3 Computing the binocular POG

The gaze point can be computed using Eq. (5) for both eyes separately and taking the middle point:


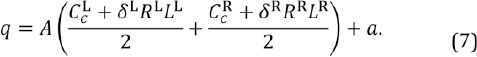


where superscripts *^L^* and *^R^* refer to left and right eye. We are left with estimating the gaze distances 5^L^ and 5^R^ for which we present two different methods. When gazing at a point, the left and right gaze vectors of the viewer are directed (approximately) to the same gaze point. Actually, due to possible fixation disparity there might be a slight mismatch between the gaze vectors (Jainta et al., 13).

We can thus minimize the squared difference between the left and right gaze points (see Eq. (4) for solving the left and right gaze distances δ^L^ and δ^R^. We get equations (8) and (9), where *g = RL/|| RL* || is the normalized gaze vector.


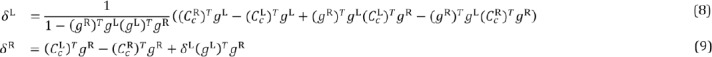


When gazing at a long distance, the gaze vectors are nearly parallel and slight inaccuracy in the estimation may cause the gaze vectors to diverge, causing the intersection to be located behind the eyes. In a simpler approach, which avoids this problem, a common gaze distance *δ = δ^L^ =* δ^R^ is estimated by assuming both gaze vectors to have equal length, resulting in a configuration where the approximated gaze vectors always cross directly on the mid- line between the eyes, in front of the nose. Here, we can define a right-angled triangle, where one of the angles is defined by the inner product of the gaze vectors, and one cathetus as half the distance between cornea centers. While in real viewing conditions where the lengths of the gaze vectors differ, the error is small as the viewing distance always clearly exceeds the distance between the eyes; in other words, the angle between the gaze vectors is typically small. For instance, when fixating a target 30 degrees to the left of the midline and one meter from the viewer, the resulting error in scene camera coordinates (located between the eyes) is only 0.03 degrees.

## 4 Fitting the physical model

As described in the previous section, the physical eye model uses pupil and cornea centers, *P_c_* and *C_c_*, for computing the POG. This section presents ways to compute these from certain features in the eye image - namely the LED reflections (that is, glints) and the pupil ellipse.

Ideally, we would like to track the 3D points *P_c_* and *C_c_* in time using, e.g., Bayesian estimation scheme for a Markov process. However, the resulting likelihood turns out to be problematic to solve as we would need to know the locations of the reflections on the surface of a sphere, projected to the 2D image plane, given locations of the light sources and the radius and center of the sphere. This appears intractable, at least in closed form and in realtime.

However, the inverse problem is solvable, that is, it is possible to compute the 3D pupil and cornea centers from the detected pupil and glint locations in the 2D image:


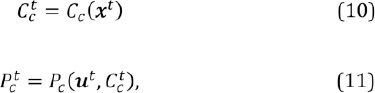


where *x* is the vector of 2D glint locations in the observed image and *u* is a set of 2D coordinates of opposing point on the pupil perimeter. The following two subsections present these computations.

### 4.1 Cornea center computation

**Figure 4:**
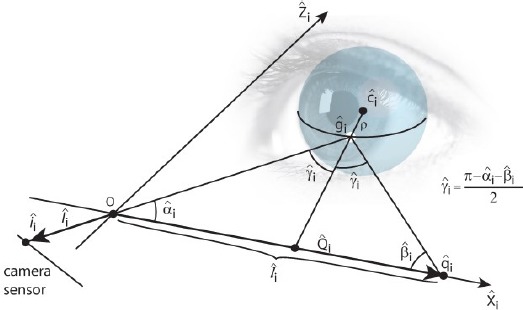
The auxiliary coordinate system, used for estimating the cornea center from glints. The origin O is at the optical center (i.e., the focal point) of the utilized pinhole camera model. Here q_L_ is the location of i’th LED light source, ĝ_i_ is the corresponding glint on the corneal sphere, *l̂_l_* is the image of the i’th glint on the image plane, and *Ĉ_l_* is the cornea center. Each LED, and the corresponding glint, has their own coordinate system with the shared origin. The figure is adapted from Hennessey et al. (10).

Computing the cornea center from the detected glints, Eq. (10), is done using a scheme that is presented by Hennessey et al. (10) and Shih and Liu (22) and explained here in brief. It is based on the law of reflection, according to which the LED source, its reflection from the corneal surface, the cornea center, camera’s optical center, and the image of the reflection on the camera’s image plane are all co-planar. Hence, we can form a glint specific auxiliary coordinate system whose origin is at the optical center of the camera, as described in Figure 4.

For the sake of clarity, the cornea center is denoted here with *c*. A light ray from *q̂_i_* reflects at *ĝ_i_* and traverses through *O* and *î_i_* Because the LED locations are assumed to be known in camera coordinates and the vectors *Î_i_* (which point from *O* to *î_i_*) are known, it is possible to compute the rotation matrix *R̂_i_* between the camera’s own coordinate system and the auxiliary coordinate system.

The cornea center can then be defined in the auxiliary coordinates as


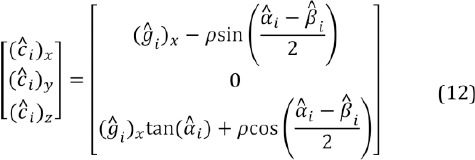


where *ρ* is the (fixed) radius of the corneal sphere and


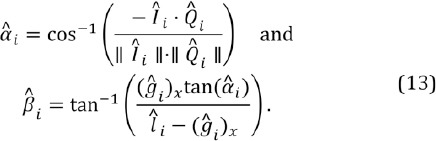


The cornea center in the auxiliary coordinate *ĉ_i_* can be transformed back to the camera coordinates *C* = *R̂^-1^_i_* *ĉ_i_*, which results in an underdetermined system of 3 equations with 4 unknowns: (*c_i_)_x_, (c_i_)_y_, (c_i_)_z_* and (*g_i_)_z_*. However, having at least two LED sources and corresponding glints provides an overdetermined system because the cornea centers equal (*c_i_*=*c_j_*∀j≠*i*) and so with two LEDs there are 6 equations and 5 unknowns. Each additional LED (and corresponding glint) increases the rank of the system so that the difference between the number of equations and unknowns behaves as *3N_L_ -* (3 *+ N_L_) = 2N_L_ -* 3 where *N*_l_ is the number of LEDs. The resulting system of (non linear) equations can be solved numerically using, e.g., Levenberg-Marquardt method.

### 4.2 Pupil center computation

**Figure 5:**
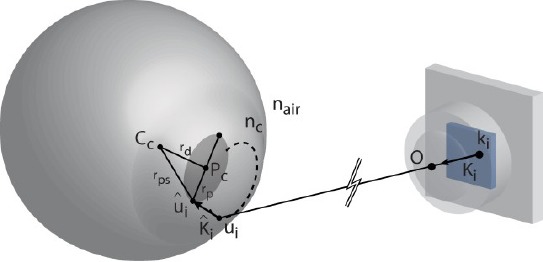
The ray tracing method for estimating the pupil center. Pc is the pupil center, Cc is the cornea center, û_i_ is a point in the pupil perimeter, u_i_ is a point in the corneal surface, K_i_ is a unit vector between these, k_i_ is the image point of u¡ , k¡ is the unit vector between these, and O denotes the optical center. The ray k¡ refracts at the cornealsurface because the refractive index of the cornea is nc > 1. The figure is adapted from Hennessey et al. (10).

In the pupil center estimation, Eq. (11), we again follow Hennessey et al.(10). The (3D) pupil center is computed as the average of different opposing points on the pupil perimeter. In order to determine the th perimeter point, a ray is traced from its image point *k_i_* on the camera’s image plane, through the optical center *O* and point *u*_i_ on the surface of the corneal sphere where the ray refracts according to Snell’s law towards the perimeter point *û_i_* (see Figure 5).

Let us first estimate the point u_i_. The origin of the camera coordinate system is at the optical center so *u_i_* can be described with a parametric equation


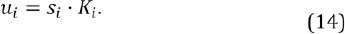


because the point u_i_ lies on the surface of the corneal sphere with center *c* and radius 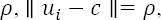 , we have a set of 4 equations with 4 unknowns(*s_i_* and the three components of *u_i_*) from which we can explicitly solve *s* and thus obtain *u_i_* .

Tracing the refracted vector *K̂* (with the known refractive index of the cornea *n_c_*) gives another parametric equation:


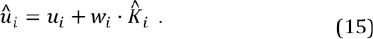


Because the distance between the pupil perimeter point and cornea center is 
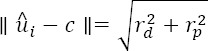
 where *r_d_* is given by population averages and *r_p_* is half of the length of the major axis of the fitted ellipse, we get a determined system of 4 equations with 4 unknowns (*w_i_* and the three components of *û_i_*) which is solvable for *w_i_*. The perimeter point *û_i_* can therefore be computed and the pupil center *P_c_* can be estimated as the average of opposing points. We use two pairs of points: the endpoints of the major and minor axis of the fitted ellipse.

## 5 Locating the pupil features

The pupil detection is relatively straightforward, utilizing conventional computer vision methods. Since the computation of the 3D pupil center requires locating at least two opposing points on the pupil perimeter, we search for an ellipse that most closely follows the pupil perimeter and use the endpoints of the ellipse axes for computing the 3D pupil center.

**Figure 6:**
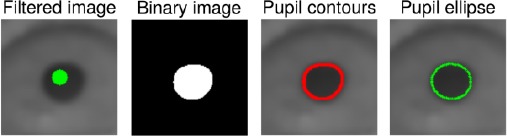
From left to right: the low-pass filtered image with the estimated pupil center (see Section 2.2); the bi- narized image; the filtered image with detected pupil contours, defined as the edges of the blob in the binarized image that contains the estimated pupil center; and the pupil ellipse that is fitted to the contours.

The opened eye image is cropped around the approximate pupil center (see Section 2.2) and low-pass filtered. The filtered image is morphologically closed to emphasize the pupil using a circular structural element with size close to the pupil size. As this may, however, disturb the pupil edges, it is heuristically summed with the non-closed image. In order to achieve invariance to average brightness of the image, the summation image is scaled so that the intensity values are in the range [0,1]. The scaled image is thresholded, using a fixed threshold value. The connected components (“blobs”) of the resulting binary image are computed, using a four-way connectivity. The blob that encloses the approximate pupil center is considered to be the pupil blob. The contours of the pupil blob are found and an ellipse is fitted to the found contours by minimizing the average distance between the ellipse and contour points (Fitzgibbon and Fisher 1995). Figure 6 gives an example of the filtered image, the binarized image, the found pupil contours, and the fitted ellipse.

Finally, in order to increase robustness against image noise, the endpoints of the major and minor axes of the fitted ellipse are filtered using the eye stability parameter *θ* (Eq. (1)) so that during the eye movements we would rely more on the measurements:


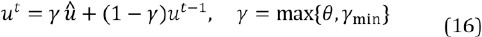


where *u* depicts any of the four endpoints and ^g^ is the corresponding measured endpoint. The thresholding of *θ* with *y_min_* is there to force the measurements to be always considered by at least small amount. This is especially important in case of smooth pursuits, during which *θ* can be too low.

## 6 Locating the cornea features

Locating the group of glints in the eye image is generally a more complex task than locating the pupil. While the glints are typically very bright in the image, it should be easy to find the possible glint pixels by their intensity values. However, the surface of the eye is approximately spherical and smooth only on top of the corneal bulge. On top of the sclera (the white of the eye), the surface is uneven and can create additional distracting and distorted reflections. Identifying true glints from the corneal surface is alleviated by the fact that the grid shape is relatively similar across eye images: the glints should approximately conform to a specific shape. In addition, during fixations the glints should appear at the same location as in the previous video frame. These three assumptions about (i) the appearance of a glint, (ii) the shape of the glint grid, and (iii) the dynamical behavior of the glints can be combined within the Bayesian framework used here. The Bayesian approach allows locating the glint grid even when some glints may be occluded or distorted due to extra reflections or the eyelid. In our previous implementation (Lukander et al., 17), the glints were identified heuristically; with the presented model grid based approach this error-prone step is avoided as an additional benefit.

### 6.1 Bayesian model for glints

Let *x* denote the glint grid, i.e., the coordinates of the glints in the eye image. The size of *x* is hence the number of LEDs, *N*_L_. The unnormalized posterior distribution for *x* under Markov assumption, is


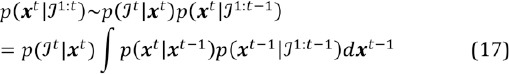


where *J^1^* denotes a “general” eye image, captured at time *t*. Due to our chosen likelihood function, we are unable to compute the integral in Eq. (17) in closed form since the (unnormalized) posterior distribution is not of a standard integrable function. The integral could be estimated with numerical methods, such as variational Bayes or Monte Carlo sampling. These methods are computationally heavy, making them unfeasible for realtime use. We thus use MAP (maximum a posteriori) estimate, that is, take the posterior to be a delta function at its maximum value:


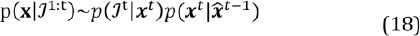


where x̂ ^t-1^ is the MAP estimate of the posterior at previous time instance. Thus the only data we have to store in memory is the previous estimate, x̂ ^t-1^, and we can write


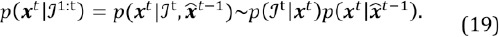


### 6.2 Implementation of the model

**Figure 7:**
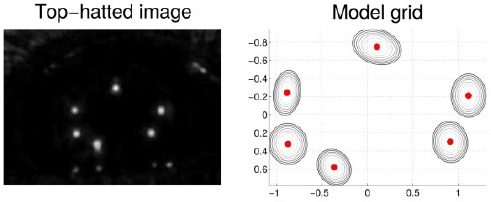
An example of a top-hat operated eye image which is cropped according to the estimated pupil center and filtered. Right: An example of a model grid. Red dots depict the mean values of the grid and the contour lines represent the covariance of each grid point. Note that in the conventional image coordinate system, the vertical coordinates increase downwards.

Section 2.2 presented cropping and opening of the input eye image and a method for finding the approximate pupil center in it. In a practical implementation of our probabilistic model for finding glints, the cropped image is filtered with the morphological top-hat operation, defined as the difference between original and opened image. The top-hatted image is cropped according to the pupil center and filtered using a small kernel to remove noise. This operated image is the input observation in the Bayes- ian model, denoted J. An example of *J* is given in Figure 7.

In our practical algorithm for finding the MAP estimate, the components are estimated one at a time. We are therefore interested on a conditional distribution, given the already estimated subset of the glint grid. The conditional posterior distribution for any component *i* of **x^t^** can be written as


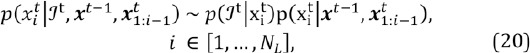


where *x^t^_1:i-1_* denotes the already estimated components of the parameter vector of which the likelihood is independent. For convenience, we have dropped the “hat” symbol in the estimate of previous *t* so that ***x**^t-1^ ≡ **x̂** ^t-1^*. Luckily, we do not need to know the normalization constant of the posterior as we are interested only on the relative values of it. Note that the dimension of a single glint *x_i_* is two as there are horizontal and vertical components. When estimating the first component, *i = 1*, there are no already estimated components: ***x1**:i-1 = **x1**:0 = {}*, and *x_1_* must be estimated by other means (our way of doing this is described in Section 6.5).

### 6.3 Likelihood model

The likelihood function should give high values for the glint locations. As these pixels are assumed to be bright (see Figure 2B), a natural form for the likelihood is


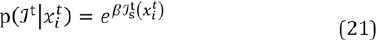


where *J _s_* is an eye image which is scaled by its maximum value: *J_s_(x) E* [0,1] ∀ *x*, and *β* is a pre-fixed parameter.

### 6.4 Prior model

Our prior assumptions are that, a priori to seeing a new image, the shape of the glint grid will be similar to what it is on average (in reality, the shape of the glint grid varies according to the position and rotation of the eye, and the individual shape of the corneal surface) and that the movement of the glints from the previous frame is in accordance with the estimated eye stability, θ. These assumptions are realized by combining two independent prior distributions, both modeled as Gaussian distributions.

The prior model for the shape utilizes a model grid which depicts how the glint grid is typically distributed, how it (co)varies, and its scale. For computing the model grid, a set of training images is collected and the glint locations are manually annotated in each image. The average grid and covariance is computed from the collected point set in a mean and scale free space which makes the system invariant to the location and scale of the eye with respect to the eye camera(s). Including rotation invariance would be useless as the pose of the glasses with respect to user’s eyes is in practice always horizontal and the rotation invariance would only increase search space and computation time. An example of a model grid is shown in Figure 7. Note that the manual annotation needs to be done only once for each device setup (not for each user).

The grid point set is denoted with *G* and its covariance with *C_G_*. The “total” prior distribution is a product of the two Gaussian distributions:


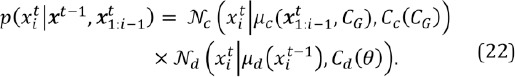


The subscript *c* stands for “conditional” and *d* for “dynamical”. The expected location of *i* th component of the conditional Gaussian, without yet taking the covariance into account, is 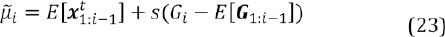 where E[.] denotes the averaging operation and *s* is the estimated scale. That is, to get the expected location of the current point (without covariance’s effect), the scaled distance of the current point of the model grid to the average of previous points of the model grid is added to the average of previously estimated grid.


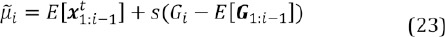


The scale *s* is estimated (when *i > 2)* as


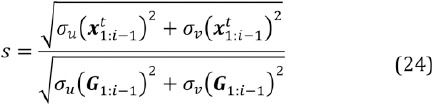


where σ_u_ denotes the standard deviation of the horizontal components of the points and *σ_ν_* that of vertical components (note that *x_t_* is two-dimensional with horizontal and vertical coordinates). Thus, if the glints appear in the eye image in same scale as in the model grid, we have *s ≈ * 1; if the eye is closer to the camera or the corneal radius is larger, compared to what was used for generating the model, we have *s > 1*. As more components are estimated (that is, increases), the uncertainty in the scale estimation decreases. For the second component ( *i =* 2), the scale is the grid model’s scale, i.e., we assume the scale to be the average scale of the training samples used for building the model grid (yet updated, see Subsection 6.6).

In order to compute the conditional mean and covari- ance, considering also the covariance *C_G_* , we partition *x*, *G*, and *C_G_* :


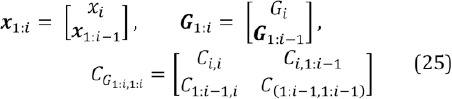


We get for the (scaled) conditional mean and covari- ance of the conditional prior distribution *N_c_*


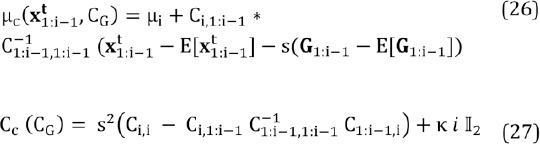


where the term *k i II_2_* regularizes the covariance (II_2_ is a *2 χ 2* sized identity matrix). This is because as *i* gets larger, the covariance gets smaller and we found in practice that eventually the prior distribution may restrict the search space too much. An alternative would be to use a prior distribution with heavier tails, such as the t-distribution.

Let us study the “dynamical” prior distribution, *N_d_*, of Eq. (22). Its mean value is simply the location of the corresponding glint in previous image:


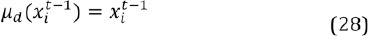


and the covariance is a diagonal matrix


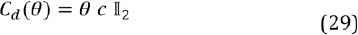


where *c* sets the upper bound for the diagonal elements. Hence, if the eyes seem to be in the same location as in the previous image based on the eye stability estimate described in Section (2.3), the glints are searched for in the close vicinity of their corresponding previous location and if the eye seems to be moving, the search space is increased. Note that *θ* ∈] 0,1 *[* and due to the ubiquitous and omnipresent image noise, *θ* is always clearly positive. However, one might want to use a lower bound on the diagonal elements of (29) to be on a safe side with its inversion.

Finally, the combined prior distribution, which is the product of two Gaussian distributions (22), is another Gaussian:


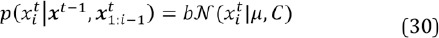


Where


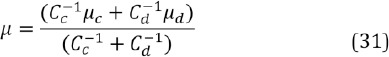


and


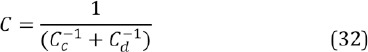


and the normalization factor is


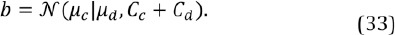


### 6.5 MAP estimation

As mentioned in Section 6.1, for the glint locations, we search for values that maximize the posterior distribution: the MAP estimate. This is done by maximizing Eq. (20) one component at a time. In order to increase robustness, instead of a single MAP estimate many estimates are searched independently by using different ordering for the components of **x^t^.** As this approach resembles Particle filtering - also known as Sequential Monte Carlo sampling (Doucet, De Freitas, and Gordon, 2) - we call the independent searches “particles” which may be considered as hypotheses for the parameter values. For examples on using Particle filtering for locating image features, see (Tamminen and Lampinen, 23; Toivanen and Lampinen, 25).

First components of the particles are initialized in the brightest pixel locations of the image *J*. The number of these “candidate” glint locations is denoted *N_glcand_*. For each such candidate location, *N_L_* particles are initialized with different component order. Hence, the number of particles is *N_part_* = *N_L_* χ *N_gl.cand_*

**Figure 8:**
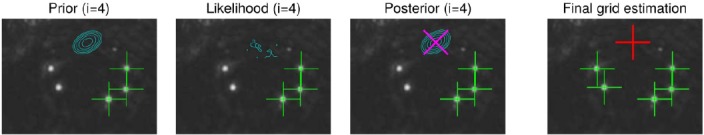
An illustrative example of locating an occluded glint. The topmost glint is occluded by the eyelid. The particle has successfully estimated three components. Since there is no visible glint where the fourth glint is supposed to be according to the prior model, the likelihood is approximately flat and the posterior equals the prior (up to a scaling factor). The remaining two glints are again successfully located. The rightmost image shows the estimated grid location. The red thick plus sign indicates a low intensity value at the corresponding glint location.

When searching the *N*_gl.cand._ brightest image pixels, the vicinity of the previously found location is overpainted so that neighboring pixels will not be chosen. The glint candidates may naturally contain also false glints but the more candidate points there are, the higher is the probability of correct glints being chosen. After each particle has searched their MAP estimates, they are compared and the one with the largest maximum value of the joint posterior (i.e., product of the MAP values of the conditional distributions) is defined as the “winner” whose parameter values are taken to be the final MAP estimate. Figure 8 exemplifies the MAP estimation procedure.

**Figure 9:**
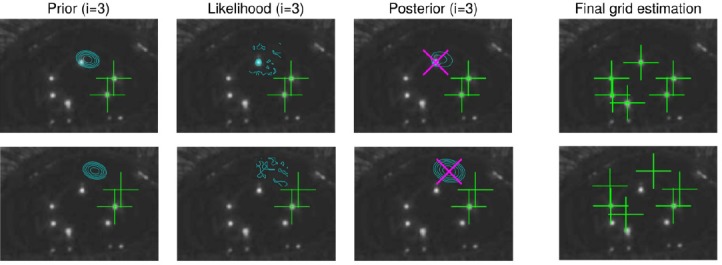
An illustrative example of the MAP estimation. Two particles are shown, in top and bottom rows. In the three leftmost panels, both particles have estimated two components, whose locations are marked with green plus signs, and are estimating their third component. The particle in the top row seems to be estimating the grid components in correct order whereas the other particle in the bottom row has a false order. The panels from left to right show respectively the contours of the prior distribution, likelihood, and posterior distribution of the third component. The distributions are evaluated only in the vicinity of the prior mean. The MAP estimate is marked with a magenta cross. The rightmost panels show the final grid estimation of the particles. The maximum of the joint posterior of the top particle is likely to greatly exceed that of the bottom row so if only these two particles were in use, the estimated glint grid location would be that of the top row particle.

**Figure 10:**
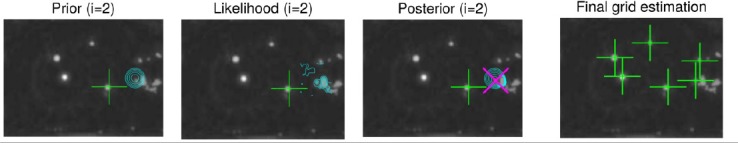
An illustrative example of finding glint when there are additional false reflections around the correct glint. The likelihood of the second glint is multimodal. The prior assumption about the glint location causes the MAP to be in the correct location. The rightmost image shows the estimated grid location.

Note that the used method always localizes the glints, whether they are visible or not. Non-visible glints are located in their supposed location as suggested by the grid model (that is, prior). Figure 9 illustrates such a case. However, when computing the cornea center (see Section 4.1), including the estimated location of the non-visible glints may increase the estimation error as opposed to including only the visible glints. Therefore, the cornea center is computed from a subset of the detected glint grid which consists of glints whose score exceeds a threshold. The score is defined simply as the intensity value at the glint in the scaled image, i.e., log likelihood (Eq. (21)) divided by β. The allowed minimum size for the subset is naturally two to be able to solve the 3D cornea center. Figure 10 gives an example of having additional distracting reflections around the “correct” glint. With a reasonable prior model, the MAP estimate is at the correct location.

### 6.6 Updating glint grid

The shape of the glint grid, ***G,*** varies from person to person, mainly due to different eye shapes. As noted, we use an average glint grid model which is a typical representation of the grid. This grid model can be modified to adapt to the personal grid by updating the mean, covari- ance, and scale recursively:


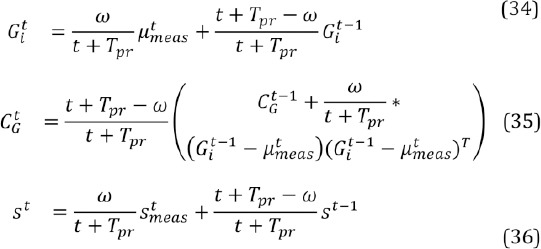


where *T_pr_* is the number of prior measurements assumed to have occured before the first frame and where the weight *ω* is defined as the logarithm of the MAP value divided by the largest possible value for the logarithm of the posterior:


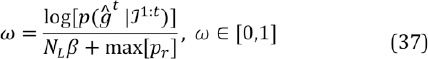


where *max[p_r_]* is the sum of the maximum values of the logarithms of the conditional prior distributions, Eq. (30), summed over the components.

## 7 Kalman filter

Despite all the advanced tracking algorithms for the glints and pupil, the estimated gaze point is often still noisy. This is understandable since even the tiniest differences in the glint locations or the endpoints of the fitted pupil ellipse cause deviation in the calculated gaze point. Because images always contain noise, the estimations of the eye features, and therefore also the gaze point, fluctuate even when steadily fixating a single point. This is at best just irritating but often disturbs the analysis and can even make the use of gaze information impractical.

As a remedy, the gaze point is smoothed by Kalman filtering which has been shown to improve performance (Toivanen, 24). A Kalman filter produces a statistically optimal closed form point estimate for the unknown state of a linear dynamic model which has Gaussian distributions for process and observation noises; see, e.g., Särkkä (21). A Kalman filter can predict the state also in case of missing observations which may happen here if the eye features of both eyes are misdetected. Comparing to prior work, Zhu and Ji (28) used a Kalman filtering for tracking the pupil and Komogortsev and Khan (15) used Kalman filter directly on the gaze signal, as is done here, but they had no observation model for the velocity component which compromises the performance with a noisy signal.

Let us denote the horizontal and vertical gaze coordinates (in the scene camera) at time instance *t* with *qU* and *qV* and their velocities with *q_u_* and *q_v_*. The movement of the gaze point is assumed to be piece-wise linear so that the state can be modeled to evolve linearly as


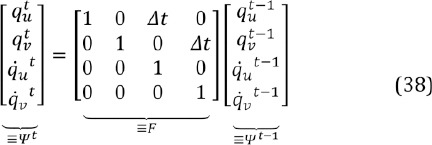


The process is assumed to be noisy with zero-mean Gaussian distribution so we get


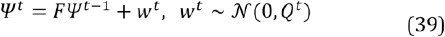


where *Q^t^* is the covariance of the process noise. The predictions for the state estimate 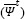 and its covariance 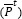 at time instance *t*, given the previous estimates 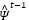 and P̂^t-1^, are


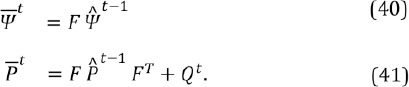


Gaze coordinates and their velocities are measured with a zero-mean Gaussian noise distribution. The observations *z^t^* at time *t* are thus related to the state by


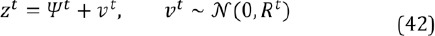


where *R_t_* is the covariance of the measurement noise. The (unknown) true state *Ψ* ^t^of the system, depicted with Equations (39) and (42), and its covariance *^U^* can be estimated as


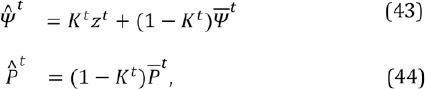


that is, the estimate for the state is a weighted average of the latest measurement and the prediction. Computing the weight as


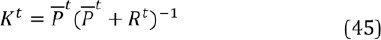


is known as Kalman filtering.

The gaze point observations are naturally the estimated gaze points, see Eq. (7). The velocity observations could be simply the derivative of the gaze point observations but then the noise in the gaze point estimates would affect the velocity estimates, too. A better approach is to utilize the eye stability parameter *θ* since it is (almost) independent on the gaze point estimates. This is beneficial especially during fixations when the pixel-wise difference in subsequent eye image frames is close to zero (⇒ *θ ≈ * 0) while the difference in gaze point estimates can be relatively large. Because *θ* only estimates the amount of movement, the direction of the velocity is computed from the gaze point estimates and the velocity observations are


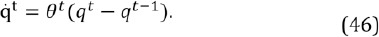


One problem with the presented Kalman filter is that the saccades are relatively sudden and fast which can lead to the assumption about the piece-wise linearity failing, especially with a low frame rate (like 30 fps). Luckily, the covariances of the process noise *(Q^t^*) and measurement noise *(R^t^*) can depend on the time instance *t*, that is, they can be modified realtime. Here the process covariance is taken to be constant, *Q^t^* ≡ *Q* ∀*t*, but the measurement co- variance is modified in realtime so that the larger the velocity estimate, the more the location observations are trusted and when the eyes seem to fixate, the location predictions are trusted more. In practice, this means that the gaze signal is filtered (almost) only during fixations; during saccades and blinks, the Kalman filtered gaze point approximately equals the “raw” gaze signal. The covariance of the measurement noise is defined as a diagonal matrix


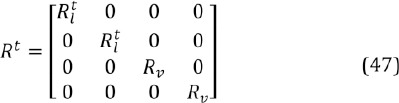


where *R_v_* is a constant variance of the velocity observation and *R_l_^t^* is a time-dependent variance of the location observation:


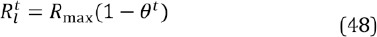


where *R_max_* is a pre-fixed maximum variance for the measurement noise. Remember that *θ ≈ 1* during and also slightly after the saccade so there is a small lag after beginning of a fixation before the signal is filtered heavier, exactly as wanted. Due to the modification of the measurement noise, the Kalman filtering causes no latency.

If the measured point is clearly outside the image area or if the measured velocity exceeds a threshold (as often happens during blinks), the measurement is considered an outlier; in this case, the prediction is used for the estimated location and the velocity is set to zero.

## 8 Algorithmic nutshell

The whole algorithm of tracking the gaze point is recapped below. The number in brackets shows the corresponding sections and subsections.

1. Preprocess eye images (2.2)
  a) Transform the images into grayscale and open and crop the images
  b) Locate the approximate pupil centers
2. Estimate the eye stability (2.3)
3. Localize glints (6)
  a) Use the likelihood and prior models (6.3, 6.4)
  b) Estimate MAP (6.5)
  c) Update the model glint grid (6.6)
4. Localize pupil (5)
5. Estimate cornea center (4.1)
6. Estimate pupil center (4.2)
7. Perform user calibration, if not already done (3.2)
8. Compute POG (3.3)
9. Kalman filter (7)

Sometimes the localization of pupil and/or glints fail (steps 3 and 4 above). In case of pupil, the failure can be deduced from the difference between the left and right pupil sizes which should approximately match. The validity of the estimated glint locations is inferred from the weights *ω*, see Eq. (37). Thresholding the weight, either of the gaze vectors can be excluded from the computation in which case the gaze point in Section 3.3 is computed using the “good” eye only and the gaze distance is estimated to be the previous successfully estimated distance. If features from both eyes are poorly localized, the frame is concluded to be part of a blink, during which the eyelid naturally occludes the pupil and glints.

## 9 Experimental evaluation

This section evaluates the performance of the presented algorithms. First, we present our hardware and software solutions, used for testing the algorithms. Next, we introduce the experimental setup, provide the used parameter values, and show some qualitative results. Then we present the performance measures which are used for reporting the numerical performance. Finally, we discuss the challenges of the system through some example cases. The study protocol has been reviewed by the Coordinating ethics committee of the Hospital District of Helsinki and Uusimaa. The recorded data is published openly

(https://github. com/bwrc/oo ga/tree/master/public data).

### 9.1 Hardware and software

**Figure 11:**
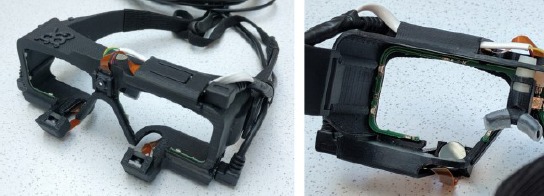
The implemented gaze tracking glasses. There are six LEDs around each eye. The scene camera is located above the nose.

For testing and utilizing the presented algorithms we have built a glasses-like 3D printed headgear where the cameras and circuit boards are attached (see Figure 11). The cameras are standard USB cameras that have no IR filters and have high pass filters inserted for blocking visible light. For both eyes there is a circuit board powering six IR LEDs, powered via the USB camera cables. As the frame is 3D printed, the positions for the cameras and LEDs relative to the cameras are extracted from the 3D model. The cameras we used capture images with VGA (640 x 480 px) resolution. The average frame rate during the experiments was 25 fps (the cameras used have a fixed iris, and the frame rate depends on available illumination). The safety issues were considered in the design so that the overall emitted radiation power is in line with the safety standard IEC 62471 (International Electrotechnical Commission 2006).

We have implemented all the presented algorithms in C++ utilizing the following libraries: The OpenCV library for computer vision tasks, the Eigen library for matrix operations, the GSL library for numerically solving the system of nonlinear equations in estimating the cornea center, and the Boost library. During the experiments, for maximum control, the software was used just to record the videos which were processed afterwards with a Dell E6430 laptop (containing i5-3320M @2.60 GHz and a Linux Ub- untu 16 LTS operating system).

### 9.2 Experimental setup

The performance of the presented system, and for reference the commercial SMI system (with iView 2.1 recording software and BeGaze 3.5 analyzing software) was tested in a laboratory setting with 19 participants (9 males, age 31 ± 8, all had normal vision with no corrective optics). The subjects sat in a chair in a dimly lit room (the lighting in the room was dim to enable automatic detection of the gaze targets in the scene video) and viewed a display while wearing the gaze tracking glasses whose camera streams were recorded. Different gaze distances were used to test performance outside the calibrated distance. The experiment included three different displays with three viewing distances (a 24” monitor at 60 cm, a 46” HDTV at 1.2 m, and a projector screen at 3.0 m) so that at each distance the resolution of the stimulus was adequate and the viewed stimuli spanned a similar visual angle.

The subjects were asked to sit relaxed and hold their head still during the measurements. User calibration was performed at only one of the three viewing distances for each subject. The presentation order of the displays and the calibration distance was permuted between the participants and each calibration distance was used equally often.

The calibration procedure cycled a stimulus dot through nine different locations on the selected calibration distance. The duration of the fixation stimuli was jittered between 2-3 s to prevent anticipatory gaze shifts. To decrease artifacts in the evaluation data, after every third location the dot changed from black to gray for three seconds signaling the subject to blink freely, while avoiding blinks during the rest of the sequence. The actual calibration of the system in this scenario was performed offline as described in Section 3.2.

**Figure 12:**
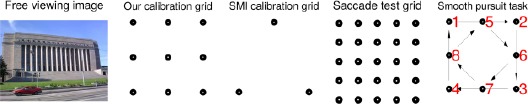
From left to right: The image presented during the free viewing task and schematic grids of the calibration, saccade, and smooth pursuit tasks (not to scale) with the starting and turning points (with running number) and movement directions of the dot in the task (i.e., the sequences are 1-2-3-4-1, jump, 5-6-7-8-5). Note that only one grid point was visible at a time.

The calibration also included a 20-second free viewing task with a picture of the Finnish Parliament House (the leftmost image in Figure 12). The stimulus was selected to supply evenly spread fixation targets across the image surface. The data recorded during free viewing was used for forming the initial grid model by manually annotating glint locations in ten eye images (per eye) for each subject. In the evaluation, a grid model was constructed from all the other subjects’ glint location data but the one being measured, as a leave-one-out scenario, to avoid overfitting. The SMI offers a 1-point and 3-point calibration routines; we used the 3-point calibration, with a triangle of dots (see Figure 12).

The test phase comprised two tasks: a saccade task and a smooth pursuit task. The saccade task included 25 stimulus locations forming a regular 5 x 5 grid - see Figure 12 - with a random presentation order (however, matching between subjects). Again, blinking was discouraged except after every third stimulus location ending with a blink pause. The smooth pursuit task presented a dot moving with a constant velocity of 3.0 degrees per second. The sequence is depicted in the rightmost panel of Figure 12. In each corner, the dot stopped for the blink pause. In each phase and for each distance, the size of the dot was one degree and the dot grid spanned an area of 24 degrees in both directions.

The performance of the presented and the SMI system was evaluated by running each stage two times, once for each system. For both devices, the calibration was performed at the first presentation distance and the same calibration information was used for all distances. The trackers were swapped during each distance and between changing distances and the order of the glasses was balanced, each system going first the same number of times.

Unfortunately, some human errors were committed during the recordings. For two subjects, the recordings for the calibration phase were accidentally deleted. In these cases, the first nine fixations in one of the other two surviving test phase recordings were used for user calibration. Additionally, for one subject the calibration display was not fully visible in the scene camera recording, inhibiting performing the calibration. One subject wore heavy mascara, challenging pupil detection for both systems, and the SMI calibration for one subject was poor. These anomalies are reported in Table 1.

The videos, recorded during the experiments, were processed using the presented method, producing gaze coor-dinates in scene camera coordinate system, the weights *ω* (see Eq. (37)), blink classification, and estimated gaze distances. If the frame was classified as a blink, it was not used in the analysis – sometimes the participants blinked also when not supposed to. The videos were also processed with the SMI software, outputting the gaze coordinates and event classification (a fixation, blink, or saccade).

### 9.3 Parameter values

The algorithms require a set of fixed parameters, tuning their performance. However, the system is not very sensitive to these as long as they are within a "reasonable" range. The parameters, their explanations and the values used are tabulated in Table 2. These values were found to give satisfactory performance during previous testing, i.e., they were not optimized in any sense for this particular da-taset. The physical eye parameters were as follows: cornea radius ρ = 7.7 mm, the refractive index of cornea *n_c_* = 1.336, and the distance between cornea center and pupil plane *r_d_* = 3.75 mm.

The most effective parameters are probably the likelihood steepness β, the prior covariance regulator κ, and the threshold parameter in the pupil detection. Computationally, the most demanding part is the MAP estimation of the glint locations. There, the computation time is directly proportional to the number of particles, i.e., the number of glint candidates *(N_glcand_)* multiplied by the number of LEDs. Therefore, we also investigate the effect of decreasing *N_glcand_*. on performance and computation time.

### 9.4 Qualitative performance

**Figure 13:**
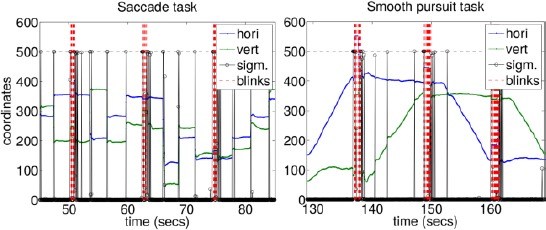
Illustrative examples of the estimated gaze tracking signal, in the scene camera coordinates. Blue and green lines show the horizontal and vertical coordinates. Estimated blinks are shown as vertical red dotted lines. The black curve shows the sigmoid values of Eq. (2), scaled between 0 and 500 which is indicated as a dashed vertical line. Left panel is from the saccade task and right panel from the smooth pursuit task.

Figure 13 exemplifies a gaze tracking signal, blink events, and sigmoid values of Eq. (2), estimated from a video material recorded during the saccade and smooth pursuit tasks. The signal seems to behave smoothly during fixations, as expected from the utilized Bayesian tracking algorithms and Kalman filtering. On the other hand, the signal succeeds to follow the saccadic behavior without an “overshooting” effect, owing to the modified measurement variance of the Kalman filter. Also the sigmoid value, which reveals the stability of the eye images, seems to behave as assumed: close to zero during fixations and unity during saccades. The signal seems reasonable during the smooth pursuits, too, albeit the signal “jerks” a bit when the stability is estimated to change. Remember that during blinks, the POG estimate is typically the Kalman filter prediction.

### 9.5 Performance measures

For an automatic performance analysis, the scene camera videos were processed with a Matlab script which automatically localizes the stimulus dot, allowing the computation of the error between the estimated and true values and thereby the numerical performance. For analyzing gaze location accuracy, after each stimulus movement a period of one second was excluded from the analysis; it was assumed that the corresponding reaction time and saccade time was never longer than one second.

As performance measures, we report accuracy and precision. Accuracy is defined as the angular error between the estimated gaze point and target point (Holmqvist et al., 11). Note that the “true” fixation target remains unknown – we can only hope that the subjects were really fixating the dot. The angle is estimated by using the known gaze distance, the metric distance between the gaze and target points, and a right triangle rule. For computing the metric distance between the points, the mm per pixel relation was estimated using known real-world dimensions of the displays and annotating the corresponding points in a few representative video frames

Precision reflects the ability of the eye tracker to reliably reproduce the same gaze point measurement and is thus related to the system noise. Good precision is desired in, at least, gaze based interaction and fixation analysis as noisy estimates during single fixation may be misclassified into several short fixations. Precision is usually defined as a root-mean-square (RMS) or root-median-square (RMedS) value of subsequent angular errors between estimated and target points, *qf^st^* and *qf^rget^*, measured during a fixation (or a separate smooth pursuit movement) (Holmqvist et al., 11). The RMS value would thus be


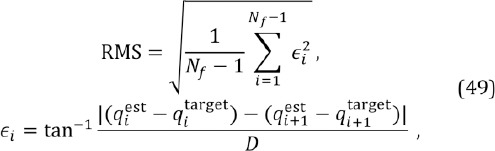


where *N_f_* is the number of fixation samples and *D* is the gaze distance (note that *q’s* are metric vectors here). RMedS would be similar but using a median value of *{ ∊_i_^2^,i = 1,…,*N_f_* — 1}* instead of mean. In addition, we report the standard deviation of the angular errors during a fixation (or smooth pursuit) as an alternative precision measure.

### 9.1 Numerical performance

**Figure 14:**
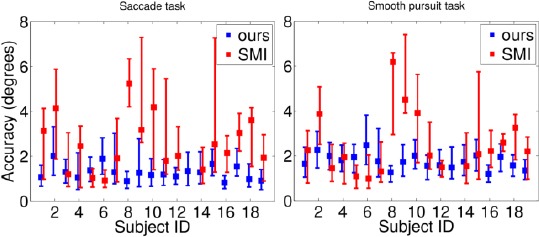
Accuracy values for the saccade task (left panel) and the smooth pursuit task (right panel) for the presented and SMI systems, using all the data. For visualization purposes, our results are slightly left and SMI’s slightly right of the tick location of the corresponding subject ID. The square depicts the median value and the endpoints of the error bars the 25th and 75th percentile values. The SMI’s accuracy values for ID13 are out of figure range. Lower values are better.

The accuracy values of each subject in saccade and smooth pursuit tasks are given in Figure 14 for both gaze trackers. The figure shows the 25th, 50th (median), and 75th percentile values of the accuracies for all three gaze distances concatenated.

. Our tracker seems to outperform the SMI system with most of the subjects. In addition, the performance of the presented system stays relatively equal for each subject, whereas SMI’s varies a lot. The SMI’s accuracies of ID13 are out of figure range, with its median values being 19 and 17 degrees for saccade and smooth pursuit tasks. In our gaze tracker, subject ID06 performs poorly due to mascara.

Evaluating a “winner” from the median values and performing a pairwise Fisher Sign Test gives a p-value of only 0.0044 for the null hypothesis that ours and SMI systems perform equally well. We can therefore conclude that in this task our system outperforms the SMI’s (P=0.0044, pairwise two-tailed Fisher Sign Test).

**Figure 15:**
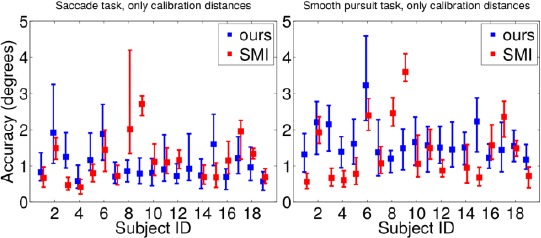
Accuracy values for the saccade task (left panel) and the smooth pursuit task (right panel) for the presented and SMI systems, using only data from the calibration distances. See caption of Figure 14 for details and note the different scale in vertical axis. Lower values are better.

A possible explanation for the difference in the performances is SMI’s apparent intolerance to the movement of the frame; based on our experiments, even slight change in locations of the cameras and LEDs with respect to the eye caused deviation in SMI’s estimated gaze point. Hence, the SMI glasses should not be moved at all once calibrated. In the experimental setup, however, the glasses were switched during each gaze distance and between them so the glasses were taken off and put back on after each distance change. The presented system is invariant to the movement of the glasses - as long as the eye camera sees the pupil and at least two LED reflections, the fixated gaze point is stationary. Figure 15 illustrates the errors only for the calibration distances, including thus only the recordings of the test phase following the calibration phase. Here the accuracy values for the SMI system show clear improvement.

**Figure 16:**
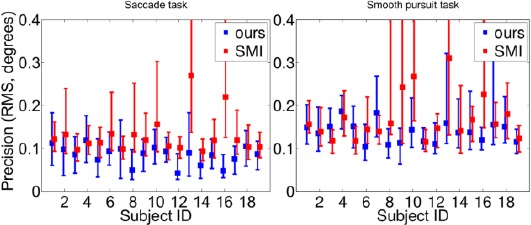
Precision values for the saccade task (left panel) and the smooth pursuit task (right panel) for the presented and SMI systems. See caption of Figure 14 for details. Lower values are better.

The precision (RMS) values are presented in Figure 16. For each subject, the RMS values of the subsequent angular errors between estimated and actual target points for each fixation are concatenated over the three gaze distances and the percentile values are computed from these. On average, the presented system provides better precision than the SMI device and the behavior is more or less similar over the subjects. For both systems, the precision is worse in the smooth pursuit task. In our case, this is because the tracking components, especially the pupil ellipse end points tracker and Kalman filtering, may lag if the eye is estimated to be stable, as is the case during a smooth pursuit.

The averaged results of all subjects are tabulated in Table 3, which shows the outstanding numerical performance of the presented system – the mean and median accuracy of the saccade task is 1.68 and 1.20 and the RMS precision is 0.12 degrees of visual angle, averaged over all the subjects and viewing distances. Dropping the anomalous test subjects, as reported in Table 1, out of the analysis improves the performance and computing the results only for the distance where the device is calibrated gives even better results; half of the time the error is less than 0.92 degrees. The probable reason for the slightly poorer performance when including also the data from other viewing distances is that the calibration scheme optimizes the correction matrix R to give best accuracy in the calibration distance – remember that R is not a pure rotation matrix and it aims to correct all error sources there are, from imperfect hardware calibration to incorrect eye parameters for the user. Still, the values of Table 3 demonstrate that the presented system performs well in all gaze distances. The performance in the saccade task is generally better than in the smooth pursuit task.

**Table 3:** The performance over all test subjects’ data for both the saccade and smooth pursuit tasks, for the presented device ("ours") and the SMI system, in degrees of visual angle. The leftmost column lists the device; next four columns report the mean, median, ω-weighted mean (see Eq. (37)), and standard deviation (STD) values of accuracy; three rightmost columns report the precision measure which are the average root-mean-square (RMS) and root-median- square (RMedS) values of subsequent angular errors between estimated and target points during fixations or a smooth pursuit movement, and an average standard deviation of the angular errors during a fixation or a separate smooth pursuit movement (STD(a)). Before computing the values, the results from all gaze distances were concatenated. The superscripts c refer to using data only from the calibration distance and * refer to excluded recordings ID06, ID07, ID11, and ID13 (see Table 1 for explanation). The values at the bottom show the average missing value rate in percentages, that is, the ratio of blinks and unavailable measurements to the number of all events. In each pair of ours and SMI results, the better result is written in italics. Lower values are better.

ACCURACY				PRECISION		
Mean	Median	W. Mean	STD	RMS	RMedS	STD(a)
Saccade task							
ours	1.68	1.20	1.63	2.21	0.12	0.07	0.38
SMI	4.09	2.42	4.09	7.89	0.35	0.20	0.68
ours^C^	1.22	0.93	1.17	1.17	0.10	0.06	0.31
SMI^C^	2.69	1.07	2.69	7.95	0.18	0.11	0.57
ours*	1.58	1.17	1.55	2.01	0.12	0.07	O.34
SMI*	3.03	2.40	3.03	2.45	0.21	0.13	O.34
ours^C^*	1.15	0.92	1.11	1.00	0.09	0.06	0.26
SMI^C^*	1.28	1.01	1.28	1.17	0.17	0.11	0.32

Smooth pursuit task							
ours	2. UI	1.67	1.94	2.05	0.26	0.13	0.73
SMI	3.52	2.28	3.52	6.53	0.54	0.28	0.89
ours^c^	1.88	1.52	1.78	2.42	0.32	0.15	0.87
SMI^C^	2.30	1.27	2.30	5.09	0.36	0.16	0.57
ours*	1.90	1.66	1.86	1.73	0.23	0.11	0.69
SMI*	2.81	2.35	2.81	2.17	0.35	0.17	O.42
ours^c^*	1.76	1.40	1.70	2.20	0.24	0.08	0.82
SMI^C^*	1.44	1.10	1.44	1.21	0.28	0.13	0.38

Average blink and missing value rate (%).							
ours				11.2			
SMI				17.4			
ours*				9.9			
SMI*				15.8			

In the third column (“W. Mean”), the POG values have been weighted by the average of left and right eye’s weights *ω* which was defined as the logarithm of the MAP value of the fitted glint grid, divided by the largest possible value for the logarithm of the posterior (see Eq. (37)). This is another benefit of the probabilistic approach: we naturally get a “score” for the estimation, reflecting the (un)cer- tainty about it. For SMI, such value is unavailable and we used unity weights there so the weighted mean equals the ordinary mean. As our weighted mean values are better than ordinary means, the *ω* seems to indeed reflect the uncertainty about the gaze point estimate and taking it into account improves the results.

As mentioned, SMI clearly suffers from the movement of the frame after calibration, resulting in decreased accuracy. However, the presented solution outperforms the SMI, in terms of accuracy and precision, also when including only the calibration measurements. The SMI performs better only in the smooth pursuit task at the calibrated distance when the “bad” subjects’ data are removed. Our system also fails on less samples (SMI 17.4 % vs. 11.2 % including all data) and while these numbers include periods during blink breaks, there is no reason to suspect that the blinking behavior would differ between the devices so our system more often produces a valid measurement.

**Figure 17:**
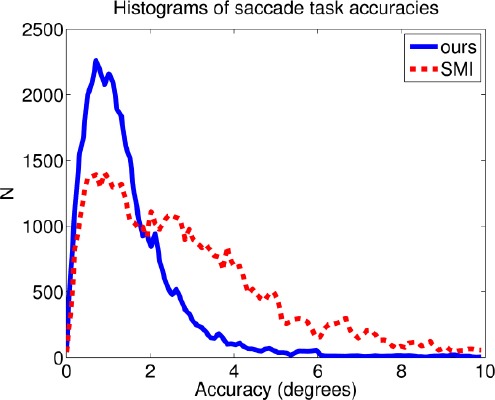
Histograms of accuracy values in the saccade task for both devices. Recordings ID06, ID07, ID11, and ID13 were excluded from the analysis. Lower accuracy value is better.

As a final comparison between the two systems, Figure 17 shows the histograms of accuracies in the saccade task, excluding the anomalous cases. Accuracy values for the presented systems are clearly concentrated around lower error values, whereas the distribution of the SMI device is wider, including values with larger errors.

Table 4 shows estimations of gaze distances with the presented system, including all the test data. The shorter distances seem to be more accurate than the largest distance whose distribution is skewed due to some very large distance estimations, manifesting also as a large difference between the mean and median values. For 3D gaze points this might present a problem. However, when the gaze distance is larger than three meters, the vergence angles of the eyes are practically in parallel and estimation errors above introduce very small changes to the 2D projected gaze point at these distances.

**Table 4:** Real and estimated gaze distances (mean, median, and STD), in meters, using all the data.

Real value	Mean estim.	Median estim.	STD of estim.
0.60	0.59	0.59	0.32
1.20	1.10	1.01	0.77
3.00	2.72	1.81	5.67

The effect of decreasing the number of glint candidates, *N_glcand_*, and thereby the number of particles in the glint finding algorithm was studied by running the results again with value *N_g_1_cand_ = 2* (corresponding to 2x6 particles) instead of *N_gicCand_ = 6* (36 particles). The computation times were estimated by processing one of the recorded videos with the two different values for *N_gicCand_*. The results are given in Table 5 and indicate that dropping the parameter value has a negligible effect on the performance but a large effect on the computation time. The lower particle number does have a slight effect on the rate of missing values as the algorithm may fail to find a good fit for the glint grid in difficult cases where only a small number of the glints are visible. Even with 36 particles, the running time of the algorithm is below 33 ms, enabling realtime handling of 30 fps camera streams, whereas using 12 particles allows to process 80 frames per second.

**Table 5:** The result of comparing two different values for N_(gl.cand.), using all the data. The columns refer to mean and median accuracy, RMS precision, missing value rate, and mean and median computation times of single frame in milliseconds. The unit of accuracy and precision is degrees. Lower values are better.

	Mean acc.	Med. acc.	RMS prec.	mv rate (%)	Mean T	Med. T
N_gl.cand._ = 6	1.68	1 .’20	0.12	11.2	30.24	29.00
N_gl.cand._ = 2	1.67	1.19	0.11	13.4	12.35	11.00

## 10 Challenges

**Figure 18:**
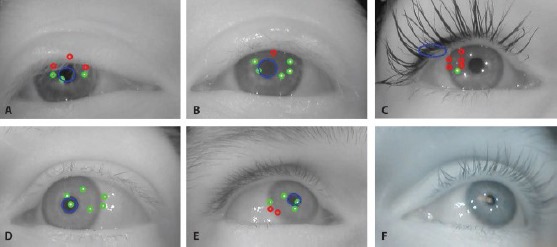
Examples of challenging issues. Green dots indicate identified glints, red dots indicate glints inferred as non-visible (at locations where the posterior glint model expects them), blue circle depicts the located pupil ellipse. (A,B) simultaneous frames for the left and right eyes, where the eyelid and eyelashes occlude some of the glints and part of the pupil. (C) Heavy mascara corrupting the pupil location and “dragging” the glint grid to the left. (D) additional false reflections on the scleral surface. (E) missing glints. (F) an example of a reflection of a window.

While the presented solution seems robust for all eyes encountered so far, the dynamic, variable nature of eye images presents occasional challenges for the algorithms. Typical examples of performance with artefactual images are presented in Figure 18: Half-closed eyelids or eyelids occluding the pupil and part of the glints as well as missing, extra, or distorted reflections from the surface of the sclera outside the corneal bulge are generally well handled. The failure of approximating the initial pupil due to, for instance, heavy mascara ruins the performance. Additionally, external IR sources such as sunlight can create extra reflections and even block some of the features - the bottom right panel of Figure 18 exemplifies this.

## 11 Discussion and conclusion

This paper has presented algorithms for tracking gaze with a mobile wearable device. The algorithms are based on a physical eye model, computer vision methods, Bayesian tracking of glints and a particle filter like method for computing the MAP estimate, binocular gaze estimation, and Kalman filtering. The main mathematical contributions are in locating the LED reflection (glints) and the pupil in the 2D eye image robustly, performing user calibration, and Kalman filtering the estimated gaze point in the 2D scene camera coordinates. The promise of the method was evaluated in experiments where 19 test subjects viewed a moving dot on three displays with different viewing distances. Publication of this experimental data is another of our contributions. Benefits of an open source publication compared to a proprietary architecture are that the full system becomes documented and the system can be modified for different needs; one can easily adjust any part of our algorithm, from image processing to system output. An additional contribution is testing the commercial SMI Eye Tracking Glasses – to the best of our knowledge, this is the first study to make a proper “scientific” quantitative evaluation of its performance.

The results show that when fixating only on the calibrated distance, our spatial accuracy is approximately one degree of visual angle; the median is slightly below, and ordinary and weighted means are slightly above one degree. When viewing also other distances – as is the case in natural viewing conditions – the accuracy deteriorates slightly. This is because the user calibration not only corrects the deviance between the measured optical vector and the “real” gaze vector, but also compensates for the other imperfections of the eye model and algorithms, and is optimal for the calibrated distance. However, the performance is good also when averaged over all the viewing distances: the median is 1.17 and weighted mean is 1.55 degrees when excluding the “anomalous” subjects. Additionally, it should be noted that the automatic localization of the target point in the calibration procedure is imperfect and the corresponding error is included in the computed error - the real error in accuracy is thus likely less than the reported values (this of course applies to SMI, too). It can be concluded that during fixations, our accuracy is better than 1.5 degrees. The precision is also fairly good: the average root-mean-square and root-median-square values of subsequent angular errors between estimated and target points during fixations are approximately 0.1 degrees. Also in the smooth pursuit task, with a moving target, the accuracy is better than two degrees.

The performance of the commercial SMI system is, in general, worse – the mean accuracy over all subjects is four degrees. This is mostly due to the intolerance of the SMI system against the movement of the device; due to the experimental setup, the device had to be removed after recording the calibrated distance. Including only the calibrated measurements gives better results but our system outperforms here, too, both in terms of accuracy and precision. Additionally, our system has a lower missing value rate. The intolerance against device movement is problematic for the SMI device in practical measurements because it necessitates monitoring the subjects during the measurements and the experiment must be aborted when the device moves and the subject needs to be re-calibrated. Due to the model-based approach, our system provides tolerance against device movement. Another nice feature of our system owes to the probabilistic approach: the estimates can be weighted by their certainties, allowing more robust estimation of the metrics that depend on the gaze point, such as the accuracy measure in our experimental evaluation.

During user calibration, the glints were sometimes misdetected and the corresponding gaze point could not be utilized in the calibration process. These were mostly some of the four corner points of the 3x3 dot grid which the subjects viewed in such a wide angle that the LEDs were reflected completely outside the corneal bulge. In retrospect, in order to ensure the quality of the calibration samples it would had been wiser to calibrate while recording, as is done in “live” usage – if the glints are not properly detected, the user is asked to move the head slightly until they are found. The user calibration scheme of SMI consisted of only three calibration points (three is the maximum) and is therefore lighter to perform than our calibration. While three points is theoretically enough for our calibration, too, the used nine points which approximately cover the viewing area give a more reliable estimate of the calibration matrix.

Computationally, the biggest effort is spent on finding glints. The computation time here is controlled by the number of particles. The results show that, at least in this setup, the performance is similar between 36 and 12 particles while the corresponding processing times were 30 and 12 ms per frame. However, in more challenging environments with distracting reflections from external sources, e.g., sunlight, the particle number may have a larger effect on the performance. Using more particles gives more robustness at the cost of computation time. In a practical implementation, the glint search process could be sped up by parallelizing each (independent) particle by making them run in their own threads or even utilizing hardware acceleration. A ruder trick would be to skip the glint and pupil estimation if the eyes seem to be fixating (and the fixation has lasted for few frames) which can be assessed from the stability parameter θ. As most of the viewing time in typical applications is spent fixating, this would speed up the computation significantly.

All algorithms and calibration routines were written in C++. Due to the involvement of the GSL library, the software is currently licensed under the GPL, version 3, while solutions for a more permissive license are sought for. The project is released in Github:

https ://github. com/bwrc/oo ga. (OOGA is Open-source GAzetracker).

## References

[1] Chen Jixu, Qiang Ji (2015). “A Probabilistic Approach to Online Eye Gaze Tracking Without Explicit Personal Calibration“. IEEE Transactions on Image Processing.

[2] Doucet Arnaud, Nando De Freitas, Neil Gordon (2001). “An Introduction to Sequential Monte Carlo Methods“. In Sequential Monte Carlo Methods in Practice.

[3] Duchowski Andrew T (2003). Eye Tracking Methodology:Theory and Practice.

[4] Evans Karen M, Robert A Jacobs, John A Tarduno, Jeff B Pelz (2012). “Collecting and Analyzing Eye Tracking Data in Outdoor Environments“. Journal of Eye Movement Research.

[5] Fitzgibbon Andrew W, Robert B Fisher (1995). “A Buyer's Guide to Conic Fitting“. In Proceedings of the 6th British Conference on Machine Vision.

[6] Guestrin Elias Daniel, Eizenman Moshe (2006). “General Theory of Remote Gaze Estimation Using the Pupil Center and Corneal Reflections.“. IEEE Transactions on Biomedical Engineering.

[7] Hales Jeremy, David Rozado, Diako Mardanbegi (2013). “Interacting with Objects in the Environment by*Gaze and Hand Gestures“*. In Proceedings of the 3rd International Workshop on Pervasive Eye Tracking and Mobile Eye-Based Interaction.

[8] Hansen Dan Witzner, Qiang Ji (2010). “In the Eye of the Beholder:A Survey of Models for Eyes and Gaze“. IEEE Transactions on Pattern Analysis and Machine Intelligence.

[9] Hayhoe Mary, Dana Ballard (2005). “Eye Movements in Natural Behavior.“. Trends in Cognitive Sciences.

[10] Hennessey Craig, Borna Noureddin, Peter Lawrence (2006). “A Single Camera Eye-Gaze Tracking System with Free Head Motion“. In Proceedings of the 2006 Symposium on Eye Tracking Research &Applications.

[11] Holmqvist Kenneth, Marcus Nyström, Richard Andersson, Richard Dewhurst, Halszka Jarodzka, Joost Van de Weijer (2011). Eye Tracking:A Comprehensive Guide to Methods and Measures. OUP Oxford.

[12] (2006). International Electrotechnical Commission.

[13] Jainta S, Hazel I Blythe, M Nikolova, MO Jones, Simon P Liversedge (2015). “A Comparative Analysis of Vertical and Horizontal Fixation Disparity in Sentence Reading.“. Vision Research.

[14] Kassner Moritz, William Patera, Andreas Bulling (2014). “Pupil:An Open Source Platform for Pervasive Eye Tracking and Mobile Gaze-Based Interaction“. In Proceedings of the 2014 Acm International Joint Conference on Pervasive and Ubiquitous Computing:Adjunct Publication.

[15] Komogortsev Oleg V, Javed I Khan (2007). “Kalman Filtering in the Design of Eye-Gaze-Guided Computer Interfaces“. In International Conference on Human-Computer Interaction.

[16] Li Dongheng, Jason, Derrick J Parkhurst (2006). “OpenEyes:A Low-Cost Head-Mounted Eye- Tracking Solution“. In Proceedings of the 2006 Symposium on Eye Tracking Research &Applications.

[17] Lukander Kristian, Sharman Jagadeesan, Huageng Chi, Kiti Müller (2013). “OMG!:A New Robust, Wearable and Affordable Open Source Mobile Gaze Tracker“. In Proceedings of the 15th International Conference on Human-Computer Interaction with Mobile Devices and Services.

[18] Mardanbegi Diako, Dan Witzner Hansen (2012). “Parallax Error in the Monocular Head-Mounted Eye Trackers“. In Proceedings of the 2012 Acm Conference on Ubiquitous Computing.

[19] Ryan Wayne J, Andrew T Duchowski, Stan T Birchfield (2008). “Limbus/Pupil Switching for Wearable Eye Tracking Under Variable Lighting Conditions“In. Proceedings of the 2008 Symposium on Eye Tracking Research &Applications.

[20] San Agustin, Javier Henrik, Skovsgaard Emilie, Mollenbach Maria, Barret Martin Tall, Dan Witzner Hansen, John Paulin Hansen (2010). “Evaluation of a Low- Cost Open-Source Gaze Tracker“. In Proceedings of the 2010 Symposium on Eye-Tracking Research &Applications.

[21] Särkkä Simo (2013). Bayesian Filtering and Smoothing.

[22] Shih Sheng-Wen, Jin Liu (2004). “ Novel Approach to 3-d Gaze Tracking Using Stereo Cameras“. IEEE Transactions on Systems, Man, and Cybernetics, Part B (Cybernetics).

[23] Tamminen Toni, Jouko Lampinen (2006). “Sequential Monte Carlo for Bayesian Matching of Objects with Occlusions“. IEEE Transactions on Pattern Analysis and Machine Intelligence.

[24] Toivanen Miika (2016). “An Advanced Kalman Filter for Gaze Tracking Signal“. Biomedical Signal Processing and Control.

[25] Toivanen Miika, Jouko Lampinen (2011). “Incremental Object Matching and Detection with Bayesian Methods and Particle Filters“. IET Computer Vision.

[26] Toivanen Miika, Kristian Lukander (2015). “Improving Model-Based Mobile Gaze Tracking“. In Intelligent Decision Technologies.

[27] Zhang Zhengyou (2000). “A Flexible New Technique for Camera Calibration“. IEEE Transactions on Pattern Analysis and Machine Intelligence.

[28] Zhu Zhiwei, Qiang Ji (2005). “Robust Real-Time Eye Detection and Tracking Under Variable Lighting Conditions and Various Face Orientations“. Computer Vision and Image Understanding.

